# Antioxidant Properties of Polysaccharide from the Brown Seaweed *Sargassum graminifolium* (Turn.), and Its Effects on Calcium Oxalate Crystallization

**DOI:** 10.3390/md10010119

**Published:** 2012-01-16

**Authors:** Chao-Yan Zhang, Wen-Hui Wu, Jue Wang, Min-Bo Lan

**Affiliations:** 1 Shanghai Key Laboratory of Functional Materials Chemistry, Research Center of Analysis and Testing, East China University of Science and Technology, Shanghai 200237, China; 2 College of Food Science and Technology, Institutes of Marine Sciences, Shanghai Ocean University, Shanghai 201306, China; Email: chyzhang@shou.edu.cn (C.-Y.Z.); whwu@shou.edu.cn (W.-H.W.); wangqilung@126.com (J.W.)

**Keywords:** calcium oxalate crystallization, antioxidant, polysaccharide, *Sargassum graminifolium*

## Abstract

We investigated the effects of polysaccharides from the brown seaweed *Sargassum graminifolium* (Turn.) (SGP) on calcium oxalate crystallization, and determined its antioxidant activities. To examine the effects of SGP on calcium oxalate crystallization, we monitored nucleation and aggregation of calcium oxalate monohydrate crystals, using trisodium citrate as a positive control. We assessed antioxidant activities of SGP by determining its reducing power, its ability to scavenge superoxide radicals, and its activity in the 1,1-diphenyl-2-picrylhydrazyl (DPPH) assay. The nucleation inhibition ratio of trisodium citrate and SGP was 58.5 and 69.2%, respectively, and crystal aggregation was inhibited by 71.4 and 76.8%, respectively. Increasing concentrations of SGP resulted in increased scavenging of superoxide anions and DPPH radicals (IC_50_ = 1.9 and 0.6 mg/mL, respectively). These results suggest that SGP could be a candidate for treating urinary stones because of its ability to inhibit calcium oxalate crystallization and its antioxidant properties.

## Abbreviations

SGP: polysaccharide from *Sargassum graminifolium*
IR: infrared spectrum
DPPH: 1,1-diphenyl-2-picrylhydrazyl free radical
Ox: oxalate
CaOx: calcium oxalate
OD: optical density
S_N_: maximum increase of optical density with time
S_A_: rate of aggregation derived from the maximum decrease in optical density
*t*_max_: maximum time
COM: Calcium oxalate monohydrate
COD: Calcium oxalate dehydrate
COT: Calcium oxalate trihydrate

## 1. Introduction

In recent years, there has been much interest in isolating novel bioactive compounds with beneficial effects on human health from marine resources. Marine algae are valuable sources of structurally diverse bioactive compounds. Sulfated polysaccharides are widespread in marine algae, especially brown seaweeds. Sulfated polysaccharides show various biological activities, including anticoagulant, antioxidant, antiviral, anticancer and immunomodulating activities [1,2,3,4,5]. Zhang *et al.* [6] reported that sulfated polysaccharides play important roles as free-radical scavengers and antioxidants, which can prevent oxidative damage in living organisms. The low molecular weight polysaccharides heparin and fucoidan provided protection against oxalate (Ox)-induced oxidative renal injury [3,7]. Ox and calcium oxalate monohydrate (COM) induce the generation of free radicals, which are major mediators of the pathologic consequences of the formation of kidney stones [8]. Hence, sulfated polysaccharides may be a potential candidate for treating urinary stones because of their protective role in Ox-mediated peroxidative injury.

Urinary stones affect a large proportion of the population. Approximately 85% of urinary stones are calcium stones, which consist of Ox and phosphate, either alone or in combination [9,10]. Crystallization is a physical and chemical process in which there is a change of state from solution to solid. This involves several physicochemical events, *i.e.*, nucleation, growth and aggregation, but the mechanisms controlling these events are not fully understood [11,12]. 

It is widely known that urinary stones frequently reoccur, and despite progress in medical therapies, there is no satisfactory drug for treatment of this condition [13,14,15]. Recent research has shown that exposure of renal cells to high concentrations of Ox and/or calcium oxalate (CaOx) crystals leads to the production of reactive oxygen species (ROS) in tissue culture and animal model studies [16,17,18]. ROS play critical roles as signaling molecules; however, an overproduction of ROS and/or a reduction in cellular antioxidant capacities due to downregulation of antioxidant enzymes results in oxidative stress [19]. Some studies *in vitro* and *in vivo* showed that treatments with antioxidants and free-radical scavengers can reduce Ox/CaOx crystal-induced injuries [18,20]. Some well-known antioxidants, such as vitamin E, have shown promising effects in populations of recurrent stone-formers [21]. These results suggest that there is great potential for the therapeutic application of antioxidants and free-radical scavengers to reduce the occurrence and reoccurrence of urinary stones and to provide superior renal protection [22]. Since the conventional treatment methods vary in their effectiveness, it is worth searching for alternative treatments, e.g., different diets or medicinal plants [8,23] for the treatment of urinary stones.

Brown seaweeds have been used in traditional Chinese medicine for more than 1000 years [24]. *Sargassum graminifolium*, a brown seaweed extensively distributed along the coasts of the South China Sea and the East China Sea, is commonly consumed as seafood, and as medical resource for its antiallergic effects [25]. To fully utilize this rich resource, it is meaningful to evaluate the polysaccharide bioactivity of *S. graminifolium*. The aim of the present study was to investigate the antioxidant properties of a polysaccharide extract from *S. graminifolium*, and to determine its ability to inhibit crystallization. These properties are useful attributes for compounds that could be used to treat urinary stones.

## 2. Results and Discussion

### 2.1. Properties of SGP

**Figure 1:**
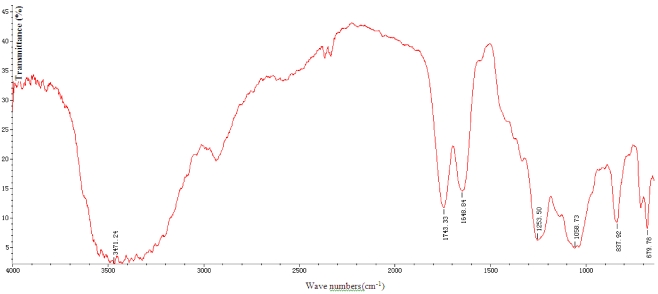
IR spectra of *Sargassum graminifolium* (Turn.) (SGP).

The results show the total sugar content is 75.68%, protein content is 0.12% and sulfate is 10.13%. Figure 1 shows that SGP in 3471, 2900, 1648 and 1253 cm^−1^ are absorption peaks, they are the stretching vibration of sugar ring O–H, C–H, C=O and S=O, so this result indicates the SGP contains high sulfate content and it is consistent with chemical analysis result.

### 2.2. Effect on Calcium Oxalate Crystallization

The inhibitory effect of SGP and trisodium citrate on CaOx crystallization is shown in Table 1. At concentrations of 4 mmol/L calcium and 0.5 mmol/L Ox, addition of 0.25 mmol/L trisodium citrate or 0.01 g/100 mL SGP resulted in a nucleation percentage inhibition ratio of 58.5 and 69.2%, respectively, and crystal aggregation was inhibited by 71.4 and 76.8%, respectively. Compared with control conditions, both SGP and trisodium citrate significantly inhibited CaOx crystallization. As shown in Table 1, addition of both SGP and trisodium citrate resulted in increases in *t*_max_ and decreased slopes of CaOx crystal growth (S_N_ and S_A_) (*P* < 0.05).

**Table 1:** Effects of SGP on calcium oxalate crystallization.

	4 mmol/L calcium/ 0.5 mmol/L oxalate	4 mmol/L calcium/ 0.5 mmol/L oxalate + 0.25 mmol/L trisodium citrate	4 mmol/L calcium/ 0.5 mmol/L oxalate + 0.01 g/100 mL SGP	*P*
*t*_max_ (min)	8.67 ± 0.94	13.44 ± 0.57	14.02 ± 0.82	*P* < 0.05 ^a^
				*P* < 0.05 ^b^
S_N_ (×10^−3^/min)	5.30 ± 1.23	2.20 ± 0.36	1.63 ± 0.28	*P* < 0.05 ^a^
				*P* < 0.05 ^b^
S_A_ (×10^−3^/min)	1.87 ± 0.62	0.53 ± 0.19	0.43 ± 0.05	*P* < 0.05 ^a^
				*P* < 0.05 ^b^

^a^ 4 mmol/L calcium/0.5 mmol/L oxalate compared with 4 mmol/L calcium/0.5 mmol/L oxalate + 0.25 mmol/L trisodium citrate; ^b^ 4 mmol/L calcium/0.5 mmol/L oxalate compared with 4 mmol/L calcium/0.5 mmol/L oxalate + 0.01 g/100 mL SGP.

### 2.3. Effect on Crystal Morphology

**Figure 2:**
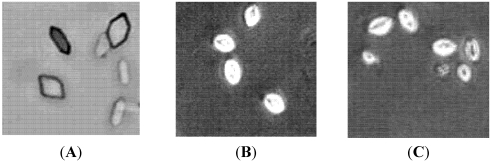
The CaOx crystals, observed under inverted microscope (100×), formed in the metastable solution of CaOx in the absence (**A**) and the presence of (**B**) trisodium citrate (1 mM) (C) SGP (0.5 mg/mL).

The amount of crystal formation, as estimated from the turbidity of the solution, is shown in Figure 2. Incubating solutions of Ca^2+^ and Ox resulted in the formation of CaOx crystals (Figure 2A) that consisted largely of hexagonal COM. Both SGP (0.5 mg/mL) and trisodium citrate (1 mM) resulted in the shape changes of CaOx crystals, as shown in Figure 2B,C; a more rounded polygonal crystals shape. This shape may prevent the formation of kidney stones, because crystals with this shape are more easily excreted in the urine compared with the COM.

Microphotography studies verified that SGP resulted in the formation of round CaOx crystals. CaOx develops in three different hydrated forms: COM, dehydrate (COD), and trihydrate (COT). COM is the most thermodynamically stable phase, followed by tetragonal COD and then triclinic COT. COM and COD are the major forms found in most urinary calculi [26,27]. SGP inhibited the growth of COM crystals, prevented the aggregation of COM crystals, and induced the formation of spherical COD crystals. These spherical COD crystals are the thermodynamically less stable phase and have weaker affinity for cell membranes than COM crystals [28]. 

### 2.4. Antioxidant Effects of SGP

**Figure 3:**
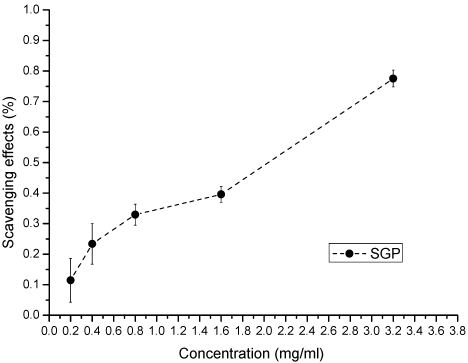
Scavenging of superoxide anion by various concentrations of SGP.

**Figure 4:**
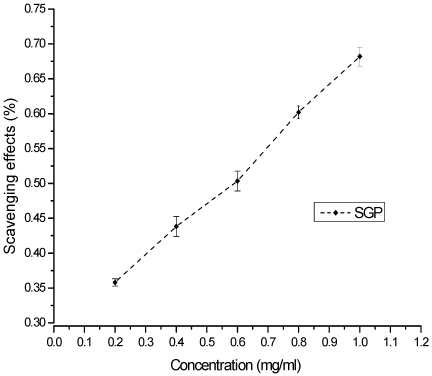
Scavenging of DPPH radical by various concentrations of SGP.

**Figure 5:**
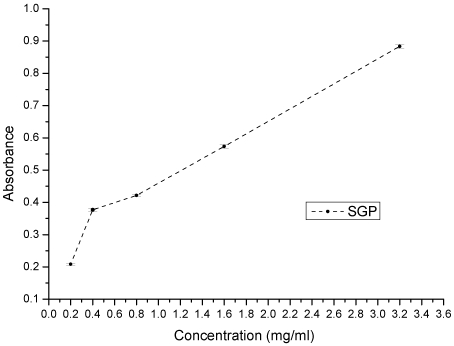
Reducing power of various concentrations of SGP.

The antioxidant properties of SGP are shown in Figure 3, Figure 4, Figure 5. Superoxide anion radicals are formed in cellular oxidation reactions, and these radicals can result in the production of hydrogen peroxide and hydroxyl radicals through dismutation and other chemical reactions. Superoxide anions have a longer lifetime and can move over greater distances than other oxygen radicals; hence, they are more damaging [29]. Therefore, the ability to scavenge superoxide anions is an important biological property for a therapeutic compound. The superoxide anion scavenging activity of different concentrations of SGP is shown in Figure 3. The scavenging effects of SGP increased with increasing concentrations; the IC_50_ was 1.9 mg/mL.

The DPPH free radical is a stable free radical that is widely used as a tool for estimating the free-radical scavenging activities of antioxidants [30]. Free radicals are highly reactive species of atoms or molecules that are unstable because of single or unbalanced electrons. DPPH is a compound with a proton free radical that has a characteristic absorption. The absorption of a DPPH solution decreases significantly in the presence of proton radical scavengers. The scavenging of the DPPH free radical by antioxidants is due to their hydrogen-donating ability [31,32]. The DPPH radical scavenging ability of SGP is shown in Figure 4. The ability to scavenge the DPPH radical increased with increasing concentrations of SGP in a concentration-dependent manner. The IC_50_ was 0.6 mg/mL. 

Reducing power assays are used to evaluate the capacity of natural antioxidants to donate an electron. Natural antioxidants are believed to break free-radical chain reactions by donating an electron or hydrogen atom to free radicals. Therefore, the reducing power of a compound is a significant indicator of its potential antioxidant activity [33,34]. The reducing power of SGP is shown in Figure 5. At concentrations of 0.2, 0.4, 0.8, 1.6 and 3.2 mg/mL, the reducing power of SGP was 0.21, 0.37, 0.42, 0.57 and 0.89, respectively. These results showed that SGP is able to donate electrons, which may be involved in its antioxidant activity.

## 3. Experimental Section

### 3.1. Chemicals and Reagents

Dry *S. graminifolium* (Turn.) was obtained from Zhuhai, Guangdong Province, China. 1,1-Diphenyl-2-picrylhydrazyl (DPPH) was purchased from Sigma Chemical Co. (St. Louis, MO, USA). All other reagents were of analytical grade.

### 3.2. Extraction of Polysaccharides from *S. graminifolium* and Its Properties

The polysaccharide extract from *S. graminifolium* (SGP) was prepared as described by Zhuang *et al.* [35]. Briefly, crude polysaccharides were extracted from the powdered seaweed by chloroform extraction, boiling water extraction, and ethanol precipitation. The degreased seaweed powder was incubated in a water bath at 90 °C for 3 h, and the residue was re-extracted twice and then concentrated to one-third of the original volume at 80 °C, adding 95% ethanol to the water extract until the ethanol concentration reached 80%. After standing overnight, the mixture was centrifuged at 2775 g for 15 min. The precipitate containing crude polysaccharides was washed with 95% ethanol, then with ethyl ether, and finally with acetone. Proteins were removed by adding trichloroacetic acid and centrifuging the mixture at 2775 g for 15 min, then added some sodium hydroxide in the centrifuged solution, to neutralize the remained TCA, and took dialysis operation for it. The resulting products were concentrated and freeze-dried, then kept it in the refrigerator.

To analyze the constituents of SGP 3 methods were used: the phenol sulfuric acid method for the determination of total sugar content, Folin-Phenol Determination method for protein content, and the barium sulfate turbidimetric method for sulfate content. Furthermore, functional groups of SGP were determined by infrared spectrum (IR).

### 3.3. Calcium Oxalate Crystallization Assay

The effect of SGR on CaOx crystallization was measured spectrophotometrically over 30 min at 620 nm. This assay quantifies crystal nucleation and aggregation in metastable solutions of Ca^2+^ and Ox. There are three parameters that characterize the crystallization process [36]: first, the maximum increase of optical density (OD) over time, termed S_N_, mainly reflects the maximum rate of formation of new particles and thus, represents crystal nucleation. Second, the rate of aggregation, S_A_, is derived from the maximum decrease in optical density. Third, the maximum time, *t*_max_, is the time at which crystals can neither nucleate nor grow. All three parameters are measurable in the crystallization process of CaOx.

We used linear regression analyses for slope measurements. The percentage inhibition was calculated from the nucleation and aggregation rates, as follows: [1 − (S_Nm_/S_Nc_)] × 100 for the rate of nucleation and [(1 − S_Am_/S_Ac_)] × 100, for the rate of aggregation (where S_m_ is the slope in the presence of the test material and S_c_ is the slope of the control experiment).

CaOx monohydrate crystallization was achieved using a mixture of calcium chloride (8 mmol/L) and sodium oxalate (1 mmol/L), containing 200 mmol/L sodium chloride and 10 mmol/L sodium acetate, adjusted to pH 5.7. The concentrations of compounds in this mixture are close to physiological urinary concentrations. The CaCl_2_ solution (1.0 mL) was stirred constantly at 37 °C both in the absence and presence of different concentrations of the test material or trisodium citrate as the positive control. After obtaining a stable base line, crystallization was induced by the addition of Na_2_C_2_O_4_ solution (1.0 mL) to reach final concentrations of 4 mmol/L calcium and 0.5 mmol/L Ox. Modifiers of CaOx crystallization were compared in assays containing 4 mmol/L calcium and 0.5 mmol/L Ox. The change in turbidity over time was measured. All experiments were run in triplicate.

### 3.4. Image Analysis of Crystal Morphology

In this study, we used imaging techniques to observe the size and morphology of the crystals and to verify the effect of incubation with the test material on CaOx crystal formation. We used stock solutions of CaCl_2_ and Na_2_C_2_O_4_ with compositions similar to those in the kinetic study. Aliquots (0.5 mL) of CaCl_2_ solutions containing SGP (0.5 mg/mL) or trisodium citrate (1 mmol/L) were added to wells in a 24-well plate. To each of the wells, Na_2_C_2_O_4_ solution (0.5 mL) was added to obtain final concentrations of 4.25 mmol Ca^2+^ and 0.75 mmol Ox [18]. Each concentration of the test material was prepared in triplicate. The plates were then incubated in a shaking water bath at 90 oscillations/min at 37 °C for 45 min. Each well was then observed under an inverted microscope (Olympus Corporation, Japan). Crystal morphology was examined in five randomly selected fields at 100× magnification. Images were captured from different fields. The most representative images are shown in Figure 2.

### 3.5. Antioxidant Assays

#### 3.5.1. DPPH Assay

The DPPH scavenging activity of the samples was measured according to the method described by Ye *et al.* [37]. Briefly, a 0.1 mmol solution of DPPH was prepared in ethanol. Then, a 2.0 mL aliquot of the DPPH solution (0.1 mM) was incubated with different concentrations of test samples (each 2.0 mL). The reaction mixture was shaken well and incubated for 30 min in the dark, and the absorbance of the resulting solution was measured at 517 nm against a blank. Measurements were performed at least in triplicate. The percentage of DPPH that was scavenged by the tested extracts was calculated using the following formula:

Scavenging effect = [1 − (A_sample_ − A_blank_)/A_control)_] × 100%

Here, ethanol (2.0 mL) plus sample solution (2.0 mL) was used as a blank and 2 mL DPPH-ethanol solution plus ethanol (2.0 mL) was used as a negative control.

#### 3.5.2. Superoxide Radical Scavenging Assay

The ability of SGP to scavenge superoxide anions was measured using a chemiluminescence method with a BPCL ultra-weak luminescence analyzer (Institute of Biophysics, Beijing, China). The chemiluminescent reaction was conducted in a Na_2_CO_3_–NaHCO_3_ (pH 10.20, 0.5 M) buffer solution. Scavenging activity of the samples was evaluated according to their quenching effects on the chemiluminescence signal of the luminal-pyrogallol system [38]. The ability to scavenge superoxide anion was calculated as follows: 

Scavenging effect = (CL_blank_ − CL_sample_)/CL_blank_ × 100%

Here, CL_blank_ and CL_sample_ represent chemiluminescence peak areas of the blank group and test group, respectively. The luminous intensity was recorded at 2-s intervals and the total luminous integrated intensity was determined for 150 s.

#### 3.5.3. Reducing Powers of SGP

Reducing powers of SGP were evaluated by the method described by Sun *et al.* [38]. Briefly, an aliquot (2.0 mL) of each sample (at different concentrations) was mixed with 2.0 mL phosphate buffer (0.2 mol/L, pH 6.6) and 2.0 mL potassium ferricyanide (1% w/v). The reaction mixture was incubated at 50 °C for 20 min, then 2.5 mL trichloroacetic acid (10% w/v) was added, and then the mixture was centrifuged at 22.2 g for 10 min. The supernatant was mixed with 2.5 mL distilled water and 0.5 mL ferric chloride solution (0.1% w/v), and the absorbance was measured at 700 nm. Increased absorbance of the reaction mixture indicated greater reducing power.

### 3.6. Statistical Analysis

All values shown are means ± SD. *P*-values less than 0.05 were regarded as significant. All statistical comparisons between groups were made using one-way analysis of variance with Dunnett’s post hoc test or by Student’s t-test. Statistical analyses were carried out using Origin 8 software.

## 4. Conclusions

The ability of urine to inhibit CaOx crystallization is an important mechanism against formation of urinary stones. Various physicochemical techniques, including turbidimetry methods, conductometric and nephelometric titrations, UV-vis and IR spectroscopy techniques, and potential measurements have been used to evaluate crystal formation [8,39]. For the current study, we used a turbidimetry method to induce and monitor crystallization because it is rapid and reproducible, and allows measurements of nucleation, growth, and aggregation of CaOx crystals [40,41]. We found that SGP inhibited CaOx crystal nucleation and aggregation at similar rates as trisodium citrate, a well-known inhibitor of CaOx crystallization that is widely used to prevent urinary stones. 

SGP has many negatively charged –OSO3–, –COO–, and –OH groups, and these anions strongly coordinate with Ca^2+^ ions [42]. The numerous negatively charged groups of SGP were able to chelate Ca^2+^ ions, resulting in a rapid increase in the concentration of Ca^2+^ ions on the surface of the SGP molecules. This resulted in a higher energy interface on the surface of SGP molecules. The adsorption of Ca^2+^ ions would result in a simultaneous decrease in free Ca^2+^ ions and an increase in the energy state of Ca^2+^ ions. Both the high energy interface and high energy state Ca^2+^ ions would then promote the formation of thermodynamically metastable COD [8]. 

Our findings indicate that natural substances such as SGP could contribute to the prevention of urinary stones. Sulfated polysaccharides from marine algae possess numerous pharmacological properties, but there is little research on sulfated polysaccharides from *S. graminifolium*. Further studies on natural substances should be carried out to assess their effects on CaOx crystallization *in vivo*.
